# Sputnik V protection from COVID-19 in people living with HIV under antiretroviral therapy

**DOI:** 10.1016/j.eclinm.2022.101360

**Published:** 2022-03-24

**Authors:** Vladimir A. Gushchin, Elena V. Tsyganova, Darya A. Ogarkova, Ruslan R. Adgamov, Dmitry V. Shcheblyakov, Nataliia V. Glukhoedova, Aleksandra S. Zhilenkova, Alexey G. Kolotii, Roman D. Zaitsev, Denis Y. Logunov, Alexander L. Gintsburg, Alexey I. Mazus

**Affiliations:** a Federal State Budget Institution “National Research Centre for Epidemiology and Microbiology named after Honorary Academician N F Gamaleya” of the Ministry of Health of the Russian Federation, Moscow, Russia; b Moscow City Center for AIDS Prevention and Control, Moscow, Russia; c Autonomous Noncommercial Organization, Moscow Center for Innovative Technology in Health Care, Moscow, Russia

## Abstract

**Background:**

HIV-infection is known to aggravate the course of many infectious diseases, including COVID-19. International guidance recommends vaccination of HIV+ individuals against SARS-CoV-2. There is a paucity of data on epidemiological efficacy assessment of COVID-19 vaccines among HIV+. This paper provides a preliminary assessment of Sputnik V vaccine effectiveness in HIV+ patients on antiretroviral therapy (ART).

**Methods:**

We performed a retrospective cohort study to assess the effectiveness of the standard Sputnik V vaccination regimen in 24,423 HIV+ Moscow residents during spring - summer 2021, that included dominance of delta variant, with estimation of hospitalization and severe illness rates in vaccinated and unvaccinated patients. Data were extracted from the Moscow anti-COVID-19 vaccination and COVID-19 incidence Registries.

**Findings:**

The data obtained indicate that Sputnik V epidemiological efficiency in the entire cohort of HIV+ on ART was 76·33%; in HIV+ with CD4+ ≥ 350 cells/µl, vaccine efficiency was 79·42%, avoiding hospitalization in 90·12% cases and protecting from the development of moderate or severe disease in 97·06%. For delta variant in this group the efficiency was 65·35%, avoiding the need for hospitalization in 75·77% cases and protecting from the development of moderate or severe disease in 93·05% of patients. There was a trend, although not statistically significant, of declining vaccine efficiency in immune-compromised individuals (CD4+ < 350 cells/µl).

**Interpretation:**

The study suggested epidemiological efficiency of immunization with Sputnik V in HIV+ ART-treated patients for the original and delta SARS-CoV-2 variants.

**Funding:**

Ministry of Health of Russia and Moscow Healthcare Department.

**Research in context**

**Evidence before this study**

We searched PubMed for research articles published up to Feb 15, 2022, with no language restrictions, using the terms “COVID-19″, “SARS-CoV-2″, “vaccine efficacy”, “people living with HIV”. We found eight peer-reviewed scientific publications, but none of them contained information on the efficacy COVID-19 vaccines in general, or on vector-based vaccine in particular on PLWH. Available data only provide insight into the immunogenicity and safety of vaccines to prevent COVID-19 in people living with HIV (PLWH).

**Added value of this study**

The data we present are the first scientific results on the preventive efficacy of a vaccine to protect against COVID-19 in PLWH. The effectiveness of the Sputnik V vaccine (adenovirus-based vector vaccine) in the group of Moscow PLWH and on ART in relation to the original strains and the Delta variant was 81.17% (49.13–93.03%) and 65.35% (52.61–74.66%), respectively.

**Implications of all the available evidence**

Our results suggest that among PLWHs with CD4+ ≥ 350 cells/µl, the standard vaccination regimen provides protection against COVID-19. Although, for patients with CD4+ < 350 cells/µl, additional studies are needed to evaluate the vaccine effectiveness and to determine the necessary frequency of immunization to achieve an acceptable level of protection, our current data provide health authorities with a basis for vaccination strategy of PLWH against COVID-19.

Alt-text: Unlabelled box

## Introduction

While various vaccines have demonstrated their high efficiency in protection of the general population from SARS-CoV-2 infection, the efficiency of these vaccines for HIV+ individuals, especially for individuals with different CD4+ T- cell counts, has still to be exhaustively evaluated. On the basis of the results of current trials, the AIDS Research Advisory Committee of the National Institutes of Health (NIH) issued interim guidance for people living with HIV (PLWH), which recommends vaccination of all patients against the new coronavirus infection, regardless of their CD4+ T-cell count or HIV RNA viral load.^1^ However, the guidance emphasizes the insufficiency of data on the efficacy of COVID-19 vaccines in the HIV+ population. Moreover, it has been reported that not all available vaccines can produce protective immunity in HIV+ people.^2^ Also, in phase three of clinical trials in which, by design, the only group representing immunosuppressive conditions were HIV+ persons, the included sample size did not achieve sufficient statistical power to evaluate the efficiency of vaccines tested in these groups.^3^, ^4^, ^5^

In the Russian Federation, 681118 PLWH were registered at territorial AIDS Centers by the end of 2019 (464·1 per 100,000 population).^6^ For these patients, SARS-CoV-2 two-injection adenovirus-based vaccine Sputnik V,^7^ registered in Russia, is widely available. The efficiency of Sputnik V against the strains circulating by the end of 2020, according to the results of clinical trials of phase 3, was 91·6%. The field efficiency in the period with active circulation of the delta variant was about 80%.^8^ This is not surprising, given that the virus-neutralizing activity against delta is 2·6 times lower than the initial level.^9^ In the HIV+ group, the efficacy of the Sputnik V vaccine is known neither for the parental strains nor for the delta variant.

Here, we provide a preliminary answer to this question. We compared the risk of SARS-CoV-2 infection in Sputnik V–vaccinated and unvaccinated HIV+ individuals at different stages of HIV disease.

## Methods

For our retrospective statistical analysis of data on COVID-19 incidence and vaccination against COVID-19 with the Gam-COVID-VAC (Sputnik V) vaccine among HIV+ people in Moscow, we obtained the data for analysis from the Moscow anti-COVID-19 Vaccination and COVID-19 incidence Registries for patients of the Moscow City Center for AIDS Prevention and Control who were receiving antiretroviral therapy (ART). The analysis used data on 24,423 patients. We assessed general and individual data on vaccine effectiveness against the original and the delta variants over selected time-periods.

Vaccine effectiveness over individual time-periods was analyzed in vaccinated and unvaccinated HIV+ individuals.

A non-immune stratum of the population was taken as unvaccinated in our calculations of vaccine effectiveness. The calculations took into account all patients in the samples, with the single exclusion of those persons not fully immunized with Sputnik V. We used a correction factor of 0·57 to calculate the non-immune stratum for the periods from January 1 to July 31 (and for the period from March 15 to May 15), and a factor of 0·54 for the period from June 1 to July 31.

To assess vaccine effectiveness in individual periods, HIV+ persons were divided into two groups: vaccinated and unvaccinated.

Individuals who received the second injection of the vaccine no later than 21 days before the end of the analysis period (before the beginning of the period and during the period) were considered fully vaccinated. (Those who fell ill within 21 days after the second shot were excluded from the analysis).

Those who were not ill (no case of COVID-19 was registered) and those who were not given a vaccine (of any kind) before and during the study period were considered unvaccinated.

A prognostic algorithm based on logistic regression model with stepwise selection of variables was designed to determine the probability of contracting COVID-19 considering the vaccination status, CD4+ T-cell level, and age and sex.

We calculated VE using the following equation:

VE = (1-RR)*100%, where RR is the risk ratio of contracting COVID-19, whether vaccinated or not.

The 95% confidence interval was calculated according to Tenny-Hoffman.^10^

In descriptions of quantitative features, we assessed the normality of the distribution by a visual method as well as by estimates of symmetry and kurtosis. To compare normally distributed features, we used the Student's T-test (the Levene test was used to evaluate the homogeneity of variants (homoscedasticity) in the groups, and Welch's T-test (for heteroscedasticity of groups). The Mann-Whitney criterion was used to compare features whose distribution differed from the normal one. The chi-square or the Fisher exact test was used to compare the qualitative variables. We carried out statistical analysis using Microsoft Excel, R and IBM SPSS Statistics version 26.

### Ethics

All information from the databases was anonymized before it was received by the research team. The study was submitted to the Local Ethics Committee of State Budgetary Healthcare Institution Infectious Diseases Hospital № 2, Moscow Health Department. The Committee concluded (Protocol № 11 of 04/10/2021) that the study does not use identifiable biological specimens and does not provide any confidential patient data. Therefore, according to the rules of the local Ethics Committee and national standards, this project does not require ethical approval.

### Role of the funding source

Ministry of Health of Russia and Moscow Healthcare Department. The funders had no role in the design and conduct of the study; in the collection, management, analysis, and interpretation of the data; in the preparation, review, or approval of the manuscript; or in the decision to submit the manuscript for publication.

Alexey I. Mazus and Alexander L. Gintsburg have accessed and verified the data and responsible for the manuscript submition.

## Results

A massive vaccination campaign of HIV+ patients undergoing treatment at the Moscow City Center for AIDS Prevention and Control was initiated in January 2021. In the first six months (from January to the end of June) of the immunization campaign, the mean vaccination rate was about 300 individuals per month, with a subsequent significant increase in vaccination in the second period in July–August to about 2000 individuals per month.

This made it possible to perform a retrospective assessment of Sputnik V efficiency in HIV+ unvaccinated and vaccinated patients undergoing ART registered at the Moscow City Center for AIDS Prevention and Control using depersonalized data (for the protocol for selection of depersonalized data from the registries, see Fig. S1). Information on vaccination and confirmed coronavirus infection was obtained from Moscow registries.

The analysis presented below covers the period from January 1, 2021 to July 31, 2021, since during this period a relatively even pace of vaccination was maintained. The cohort for this study included individuals who received the second dose of the vaccine no later than July 10, 2021 (21 days before the end of the period of analysis) as fully vaccinated (21 days are required to achieve full protection). Those receiving the second injection of the vaccine after July 10 were excluded from the calculations, but not from the entire sample. Patients receiving the first injection after July 31, or not receiving the vaccine, were considered unvaccinated.

Diagnoses of COVID-19 in patients were based on clinical examination, epidemiological anamnesis, positive laboratory tests (PCR, ELISA), and/or chest CT. Histories of COVID-19 infection prior to the specified time period were not excluded from the calculations, following adjustments on population immunity. Cases of COVID-19 infection after the specified period were not included in the final analysis.

After application of above-mentioned exclusion criteria, our cohort consisted of 24,423 patients. Characteristics of patients of this cohort are shown in Table 1. 10·4% of HIV+ individuals were fully immunized by receiving two injections of the vaccine during the specified period. The mean ages of patients did differ significantly in the subgroups analyzed. In general, HIV+ people usually represent a rather heterogeneous group of patients, and therefore, to assess the effectiveness of vaccination, it is certainly important to take into account the status of their immune system. Therefore, we monitored CD4+ T-cell counts. Data on CD4+ T-cell counts were available only for 17,885 patients. Vaccinated patients had generally higher levels of CD4+ T cells.

**Table 1:** General characteristics of HIV+ individuals from the Moscow COVID-19 Vaccination and COVID-19 incidence Registries (patients on file at AIDS Moscow City Center).

Sample characteristics	Vaccinated, *n* = 2543 (10·4%)	Unvaccinated, *n* = 17,592 (72·0%)	Uncompleted vaccination *, *n* = 4288 (17·5%)	*p*
Mean age (*M*±SD, 95% CI)	44·69 ± 10·12 (44·30–45·09)	41·50 ± 10·11 (41·35–41·65)	41·65 ± 8·28 (41·40 - 41·90)	<0·001 Welch's T-test)
Females	762 (30·0%)	7883 (44·8%)	1798 (41·9%)	<0·001 (chi square)
Males	1781(70·0%)	9709 (55·2%)	2490 (58·1%)	
CD4+ (Me[IQR]) cells/µl (*n* = 17,885, 73·2%)	*n* = 2198 (86·4%) 639[484 - 821]	*n* = 12,134 (69·0%) 526[334 - 730]	*n* = 3553 (82·9%) 586[424 - 774]	<0·001 (Mann - Whitney test)
СD4+ ≥ 350 cells/µl (*n* = 13,846)	1967 (89·5%)	8883 (73·2%)	2996 (84·3%)	<0·001 (chi square)
СD4+ < 350 cells/µl (*n* = 4039)	231 (10·5%)	3251 (26·8%)	557 (15·7%)	

⁎ data of patients with incomplete vaccination were not considered in group comparison.

The risk of contracting COVID-19 was assessed in the subgroups of HIV+ patients with different immune statuses (Table 2). The analysis shows that full immunization with Sputnik V in patients with counts CD4+ T-cell ≥ 350/µl resulted in a 3·29-fold (95% CI 2·51–4·30) reduction of COVID-19 infections compared with unvaccinated ones. In the subgroup of patients with a compromised immune function (CD4+ T cells < 350/µl), vaccination reduced the risk of COVID-19 by 2·53 times (95% CI 1·40–4·60). For both subgroups, the risk reduction for contracting COVID-19 was statistically significant (*p* < 0·002) (Table 2).

**Table 2:** Characteristics of patients receiving ART in subgroups by CD4+ T*-*cells count (only fully immunized and unvaccinated were considered).

Patients’ status	СD4+ ≥ 350 cells/µl			СD4+ < 350 cells/µl		
	Vaccinated	Unvaccinated	*P* < 0·001 OR = 3·29 (2·51–4·30)	Vaccinated	Unvaccinated	*P* = 0·002 OR = 2·53 (1·40–4·60)
With prior history of COVID-19	51 (2·6%)	779 (9·3%)		11 (4·8%)	352 (11·7%)	
No prior history of COVID-19	1916 (97·4%)	7573 (90·7%)		220 (95·2%)	2645 (88·3%)	

Analysis of COVID-19 severity in the vaccinated subgroup shows that fractions of cases with no lung tissue damage (CT 0) in HIV+ patients with CD4+ T-cell counts ≥ 350 cells/µl was significantly higher than in the matching unvaccinated subgroup (*p* = 0·043). In general, mild or moderate lung tissue damage, as evaluated from chest CT imaging (CT 0–2), was observed in 95·3% of unvaccinated patients with preserved immunity, while severe and critical lung tissue damage (CT 3–4) was registered in approximately 4% of cases. In contrast, among unvaccinated HIV+ patients with low CD4+ T-cell counts severe lung tissue damage (CT 3–4) was documented in 17·6%, as shown in Table 3: more than 4 times common than in patients with CD4+ T-cell counts ≥ 350 cells/µl. It is important to mention that in severely immunodeficient HIV+ patients, lung tissue damage caused by opportunistic pathogens is almost indistinguishable from lesions caused by the SARS-CoV-2 virus. Therefore, radiological evaluation in these patients is usually challenging in a syndemic context.

**Table 3:** Typical for COVID-19 chest CT imaging features with extents of pulmonary involvement in HIV+ on ART depending on СD4+ Т-cells count.

	СD4+ ≥ 350 cells/µl			СD4+ < 350 cells/µl		
	Vaccinated (documented *n* = 15)	Unvaccinated (documented *n* = 334)	*P*	Vaccinated (documented *n* = 6)	Unvaccinated (documented *n* = 228)	*P* (Fischer's Exact test)
**CT 0**	7 (46·7%)	76 (22·8%)	0·043	1 (16·7%)	55 (24·1%)	0·916
**CT 1**	6 (40·0%)	197 (59·0%)		4 (66·7%)	94 (41·2%)	
**CT 2**	0 (0·0%)	45 (13·5%)		1 (16·7%)	39 (17·1%)	
**CT 3**	2 (13·3%)	13 (3·9%)		0 (0·0%)	25 (11·0%)	
**CT 4**	0 (0·0%)	3 (0·9%)		0 (0·0%)	15 (6·6%)	

Both among the general population of Moscow residents and among ART-treated HIV+ subpopulations on file at AIDS Moscow City Center, around 11% were diagnosed with COVID-19 since the pandemic outbreak and until early July 2021. This suggests that the strata of the SARS-CoV-2 contacts in these groups will be comparable. According to the Moscow City Health Department, the entire stratum of individuals immune to SARS-CoV-2 reached 43% in April and 46% in June 2021. All patients were included in the analysis to calculate vaccine effectiveness in comparison with unvaccinated cohorts, except for individuals with incomplete immunization.^11^

The analysis shows that the overall epidemiological effectiveness of vaccination with Sputnik V in HIV+ patients undergoing ART included in our study was 76·33% (95% CI 69·84–81·43%) (Table 4).

**Table 4:** Overall vaccine effectiveness in the entire group of HIV+, receiving ART.

Patient cohorts	Vaccinated	Unvaccinated
COVID-19 illness	71 (2·8%)	1354 (8·2%)
No Covid-19 illness	2472 (97·2%)	15,252 (91·8%)
Prior history of Covid-19	0	986
Excluded from calculation due to incomplete immunization	4288	
Epidemiological effectiveness	76·33% (95% CI: 69·84% - 81·43%) *р* < 0·001 (chi square test)	

The effectiveness of the vaccine depended on the immune status of the individual: In the group of patients with CD4+ T-cell counts ≥ 350 cells/µl, the effectiveness was higher and constituted on average 79·42% (95% CI 72·54–84·57%), while in the group with CD4+ T-cell counts < 350 cells/µl it was lower, on average 73·15% (95% CI 50·27–85·50%) (Table 5). Therefore, vaccine effectiveness in HIV+ on ART with preserved immune status (CD4+ T-cell counts ≥ 350 cells/µl) was not different from the 80% level in the general population.^7^^,^^8^

**Table 5:** Vaccine effectiveness among HIV+ in subgroups by CD4+ counts.

CD4+ count	CD4+ < 350, *n* = 4039			CD4+ >=350, *n* = 13,846		
Patient cohorts	Vaccinated documented (*n* = 231)	Unvaccinated documented (*n* = 2997)	P (chi square test)	Vaccinated documented (*n* = 1967)	Unvaccinated documented (*n* = 8352)	P (chi square test
COVID-19 illness	11 (4·8%)	352 (11·7%)	0·002	51 (2·6%)	779 (9·3%)	<0·001
No Covid-19 illness	220 (95·2%)	2645 (88·3%)		1916 (97·4%)	7573 (90·7%)	
Prior history of Covid-19	0	254		0	531	
Excluded from calculation due to incomplete immunization	352			779		
Epidemiological effectiveness	73·15% (50·27–85·50%)			79·42% (72·54–84·57%)		

Significantly fewer hospitalizations due to COVID-19 (*p* = 0·041, chi-square test) were documented for vaccinated patients with CD4+ ≥ 350 cells/µl, whereas for patients with compromised immune status, the trend to reduction did not reach statistical significance (*p* = 0·358, Fisher's exact test).

Continuous monitoring of SARS-CoV-2 virus variability in Moscow suggests a logical division of analyzed data into two parts – the spring (March–May 2021) period with predominance of original SARS-CoV-2 (the first delta case was documented in April 2021), and the summer months (June–July 2021), when delta and subsequent variants became predominant.^12^^,^^13^ This approach allowed us to distinguish the effectiveness of the Sputnik V vaccine in HIV+ people in Moscow against the original SARS-CoV-2 and the delta variants.

For differential assessment of vaccine effectiveness against the original and the delta variants in HIV+ people, two virtual samples were formed. The first follow-up period included events from March 15 to May 15. During this period, patients who received both injections of the vaccine before April 24 inclusive were considered vaccinated, and patients who did not receive any component of the vaccine before and during the follow-up period were considered unvaccinated. Patients who had incomplete immunization, with the first injection but not the second by April 24, were excluded from the calculation. Unvaccinated patients and patients with a prior history of COVID-19 regardless of their immunization status were included in the calculation, with subsequent adjustment for population immunity (43% of the population having antibodies) to assess the effectiveness of the vaccine.

The second period included all cases followed up during June 1–July 31. During this period, patients who received both components of the vaccine up to July 10 inclusive were considered vaccinated. Similar criteria and a similar approach to assessment of the non-immune population (accounting for 46% having antibodies) were used in the second period. Sex-related significant differences between vaccinated and unvaccinated patients were documented in these two samples, just as in the entire study population. Characteristic features of the two samples are presented in the appendix (Table S1).

The study suggested that the epidemiological effectiveness of Sputnik V vaccination in the first time-period was 71·74% (95% CI 42·85–86·03%), and in the second period was 59·77% (95% CI 47·28–69·30%) (Table 6).

**Table 6:** Overall epidemiological effectiveness of vaccination against original and delta variants.

Period	15 March–15 May (circulation of original variant)	1 June–31 July (circulation of delta variant)
Number of vaccinated with *no prior history* of COVID-19 infection	1257	2543
Number of unvaccinated with *no prior history* of COVID-19 infection	21,193	15,882
Number of COVID-19 breakthrough cases among vaccinated, *n*	8	59
Number of COVID-19 cases among unvaccinated, *n*	305	630
Number of total COVID-19 cases before the beginning of analyzed period (regardless the immunization status)	1308	1722
Number of excluded from calculation due to incomplete immunization	665	4288
VE,% (95% CI)	71·74% (42·85–86·03%)	59·77% (47·28–69·30%)

Of note is the fact that estimated vaccine effectiveness in the subgroup of patients with CD4+ T-cell counts ≥ 350 cells/µl was 81·17% (95% CI 49·13–93·03%) and 65·34% (95% CI 52·61–74·66%) against the original and delta variants, respectively. Whereas in patients with CD4+ T-cell counts < 350 cells/µl, vaccine effectiveness was 33·47% (95% CI -113·50–79·27%) and 55·05% (95% CI 2·59–79·26%) against the original and delta variants, respectively, a finding that is difficult to interpret because of the lack of statistical power and the width of the confidence interval (Table 7).

**Table 7:** Impact of patients’ immune status on vaccine effectiveness during two time periods.

Time period	15 March–15 May		1 June–31 July	
Immune status of HIV+ on ART	CD4+ < 350 cells/µl	CD4+ ≥ 350 cells/µl	CD4+ < 350 cells/µl	CD4+ ≥ 350 cells/µl
Number of vaccinated with *no prior history* of COVID-19 infection	111	961	227	1961
Number of unvaccinated with *no prior history* of COVID-19 infection	3501	116,761	2774	7961
Number of COVID-19 breakthrough cases among vaccinated, *n*	3	4	7	45
Number of COVID-19 cases among unvaccinated, *n*	92	168	129	388
Number of total COVID-19 cases before the beginning of analyzed periods (regardless the immunization status)	361	695	477	922
Number of excluded from calculation due to incomplete immunization	66	514	557	2996
VE,% (95% CI)	33·47% (-113·50%–79·27%)	81·17% (49·13%–93·03%)	55·05% (2·59%–79·26%)	65·34% (52·61–74·66%)

Analysis of vaccine effectiveness in terms of prevented hospital admissions and protection against disease progression to moderate or severe forms of COVID-19 shows that in patients with CD4+ T-cell counts ≥ 350 cells/µl the vaccine averted hospitalization in 100% of the group during the first period and in 75·77% (95% CI 44·25–89·47%) during the second (with predominant occurrence of the delta variant). In immunodeficient patients, these percentages were 64·82% (95% CI -156·32–95·17%) and 59·92% (95% CI -28·74–87·52%), respectively (Table 8).

**Table 8:** Impact of patient's immune status on vaccine effectiveness in terms of protection from hospitalization during two time periods.

Time period	15 March - 15 May		1 June–31 July	
Immune status of HIV+ on ART	CD4+ < 350 cells/µl	CD4+ ≥ 350 cells/µl	CD4+ < 350 cells/µl	CD4+ ≥ 350 cells/µl
Number of vaccinated with *no documented COVID-19 illness*	111	961	227	1961
Number of unvaccinated with *no documented* COVID-19 illness (including immune stratum)	2201	7051	1755	4796
Number of hospitalized among vaccinated, *n*	1	0	3	6
Number of hospitalized among unvaccinated, *n*	58	54	62	74
VE,% (95% CI)	64·82% (-156·32–95·17%)	100%	59·92% (-28·74–87·52%)	75·77% (44·25–89·47%)

Vaccine effectiveness in preventing moderate or severe infection in patients with relatively preserved immune function was 100% in March–May 2021 and 93·05% (95% CI 49·51–99·04%) in summer 2021. The corresponding numbers in patients with CD4+ T-cell counts < 350 cells/µl were 27·14% (95% CI -440·48–90·18%) and 38·64% (95% CI -159·75–85·51%), respectively, which probably reflects insufficient information because of the width of the confidence interval (Table 9).

**Table 9:** Impact of patient's immune status on vaccine effectiveness against severe disease during two time periods.

Time period	15 March–15 May		1 June–31 July	
Immune status of HIV+ on ART	CD4+ < 350 cells/µl	CD4+ ≥ 350 cells/µl	CD4+ < 350 cells/µl	CD4+ ≥ 350 cells/µl
Number of vaccinated with *no documented COVID-19 illness*	111	961	227	1961
Number of unvaccinated with *no documented* COVID-19 illness (including immune stratum)	2201	7051	1755	4796
Number of hospitalized among vaccinated, *n*	1	0	2	1
Number of hospitalized among unvaccinated, *n*	28	32	27	43
VE,% (95% CI)	27·14% (-440·48–90·18%)	100%	38·64% (-159·75–85·51%)	93·05% (49·51–99·04%)

We designed a prognostic model to determine the probability of contracting COVID-19 based on the vaccination status, CD4+ T-cell level, and age and sex of patients. We used a logistic regression model with stepwise selection of variables. Predictors were excluded at *p* values > 0.05. The dependent probability of contracting COVID-19 is described by the equation: *p* = 1 / (1 + e-z) * 100%. *z* = -3·953 + 0·007*X_age_ – 1·290*X_vaccination_ + 0·275*X_CD4+_ + 0·155*X_gender_, where: *p* is the probability of contracting COVID19 during the analyzed period (%), X_age_ is the age (full years), X_vaccination_ is the–vaccination status (0, not vaccinated;1, vaccinated), X_CD4+_ is the CD4+ T-cell count (0, < 350; 1, ≥ 350), X_gender_ is sex (0, Male; 1, Female).

The resulting regression model is statistically significant (*p* < 0·001); however, according to the Nigel Kirk determination coefficient, the model takes into account only 2·9% of all factors determining the probability of contracting COVID-19 (Fig. S2). A 1-year increase in the age variable, holding all other variables constant, increases the chance of contracting COVID-19 by a factor of 1·007 (95% CI: 1·001–1·014). Being vaccinated with other variables fixed decreases the probability of contracting COVID-19 by 3·631 times (95% CI: 2·794–4·720) as compared with being unvaccinated. A CD4+ T-cell count ≥ 350 decreased the probability of contracting COVID-19 by 1·317 times (95% CI: 1·155–1·502). Being a woman increases the probability of COVID-19 by a factor of 1·168 (95% CI: 1·035–1·317) as compared with men, with the other variables fixed. There is a positive correlation between the probability of contracting COVID-19 and age, and a negative correlation with CD4+ T-cell count and being vaccinated. Women have a higher risk of contracting COVID-19 than men.

## Discussion

A number of vaccines for prevention of COVID-19 have been developed, which have proven highly effective in a population of relatively healthy individuals during clinical trials.^3^, ^4^, ^5^^,^^14^ Unfortunately, the effectiveness of these vaccines against the delta genetic lineage of SARS-CoV-2 was lower,^15^^,^^16^ therefore explaining, in part, increasing global incidence of COVID-19 even in highly vaccinated populations.^17^ Nevertheless, even in the case of delta variant infections, these vaccines largely retained their effectiveness against severe disease and against the necessity for hospital admissions in the general population, justifying the further use of vaccines against COVID-19.

The immunogenicity of COVID-19 vaccines in HIV+ people with CD4+ T*-*cell counts > 250 cells/µl was reported.^18^, ^19^, ^20^ However, there is still a lack of data on vaccine effectiveness in preventing severe disease and deaths among HIV+ people (in particular who are infected with the delta SARS-Co V-2 variant). Here, we report on the effectiveness of Sputnik V vaccine in HIV+ people, with standard prime-boost dosing with a three-week interval between the two injections.

To evaluate the effectiveness, we used data on HIV+ people from the Moscow anti-COVID-19 vaccination and COVID-19 incidence Registries. The study included 24,423 patients of the Moscow City Center for AIDS Prevention and Control receiving ART. Only people with two vaccine doses have been included. The two-dose vaccine was a standard protocol tested in a clinical trial and allowed to be used for COVID-19 prevention.^9^^,^^14^ These data allowed us to estimate Sputnik V vaccine effectiveness for PLWH under ART depending on their immune status. In individuals with CD4+ T*-*cell counts ≥ 350 cells/µl, the effectiveness of the vaccine was not different from that in uninfected people. It prevented infection, helped to avoid hospitalization (almost by 100%) and death. Basically, vaccine effectiveness in HIV+ persons on ART with preserved immune status (CD4+ T*-*cell counts ≥ 350 cells/µl) was not very different from the 80% level in the general population.^9^^,^^14^^,^^21^ As with uninfected individuals, the effectiveness of the vaccine against the delta variant was lower than against earlier variants.

Therefore, the data obtained indicate that Sputnik V is effective in HIV+ individuals with CD4+ T*-*cell counts ≥ 350 cells/µl. Vaccination significantly reduces the risks of becoming severely ill and of requiring hospitalization. It is important that the effectiveness of protection against the delta strain was maintained at a high level. In patients with CD4+ T*-*cell counts < 350 cells/µl, vaccine effectiveness was lower but was still present. There were insufficient data to reliably calculate the effectiveness of the vaccine in this group. Better protection can be achieved after additional dose-testing and adjustment of immunization schedules in this patient population. Our results show that treatment of HIV leading to CD4+ T*-*cell reconstitution is beneficial for infected individuals and also provides a community benefit by decreasing the spread of other infectious agents including SARS-COV-2. This relationship among pathogens is well known for other agents, in particular tuberculosis.^22^

Our study has several limitations: (i) The study groups were not sufficiently homogeneous (the vaccinated group included significantly more males and 16% more patients with CD4+ T*-*cell counts > 350 cells/µl, and was on average three years older than persons in the unvaccinated group; (ii) The sample size for immunocompromised patients was not sufficient to estimate the exact level of effectiveness of vaccine protection for this subgroup; (iii) The data were insufficient for a number of parameters, resulting in some cases excessively wide confidence intervals; (iv) Factors such as BMI, comorbidities and ethnicity were not taken into account due to the lack of these data, which may affect the residual bias in our regression model.

We did not observe any neurological or inflammatory disorders in HIV+ patients vaccinated with Sputnik V. The adverse events profile we observed was limited to local injection site reactions such as pain, redness, and swelling and to general reactions such as weakness, malaise, muscle ache, headache, and fever. However, given that our study was not specifically focused on assessment of rare adverse events. This is another limitation of our study.

With the widespread prevalence of virus variants of concern, the duration of the protective effect of vaccines is reduced, creating the need for revaccination. So, after the appearance of delta, most developed countries have introduced revaccination programs.^23^ In the case of HIV +, it is not clear how long the protection can be achieved even with the initial two-dose vaccination. It cannot be ruled out that in the HIV+ group the achieved protective period will be shorter. The data set we were analyzing does not allow us to draw conclusions about the dynamics in the protective effect of Sputnik V. Such analysis will be made subsequently after accumulation of a sufficient amount of data on the HIV+ population in the follow-up.

Nevertheless, we believe that our study allowed us to evaluate the effectiveness of vaccination with Sputnik V against COVID-19 in the group of HIV+ people receiving ART, and thus is important for the development of vaccination policy for such individuals.

The results obtained suggest that Sputnik V vaccine is efficient in protection of HIV+ Moscow residents under ART from SARS-CoV-2 infection, especially from the most severe effects of COVID-19: the need for hospitalization, and death. However, in immunocompromised HIV+ individuals vaccine effectiveness was lower than in non-immunocompromised HIV+ patients. The effectiveness of this vaccine against the delta variant of SARS-CoV-2 was only slightly lower than against the original variant in patients with CD4+ T*-*cell counts > 350 cells/µl. In summary, despite decreased epidemiological effectiveness against the delta variant especially in immunocompromised HIV+ individuals undergoing ART, Sputnik V vaccine protection against moderate or severe disease remains sufficient for it to be recommended it for all HIV+ ART-treated individuals. A similar analysis should be now performed for the upcoming omicron SARS-CoV-2 variant.

## Declaration of interests

ALG, DYL, DVS have patent pending for the Sputnik V immunobiological expression vector, pharmaceutical agent, in COVID-19 research. The patent owner is the “National Research Center for Epidemiology and Microbiology named after Honorary Academician N F Gamaleya” of the Ministry of Health of the Russian Federation (Moscow, Russia). All other authors declare no competing interests.

## Appendix Supplementary materials

Image, application 1

Image, application 2
