# Supplementary material for: Sputnik V protection from COVID-19 in people living with HIV under antiretroviral therapy
Source: eClinicalMedicine. 2022 Mar 24;46:101360. doi: 10.1016/j.eclinm.2022.101360 (PMC8943473; doi:10.1016/j.eclinm.2022.101360)
Supplement: Supplementary file 1 [file mmc1.docx]

**Figure S1. Database-processing algorithm.**

**
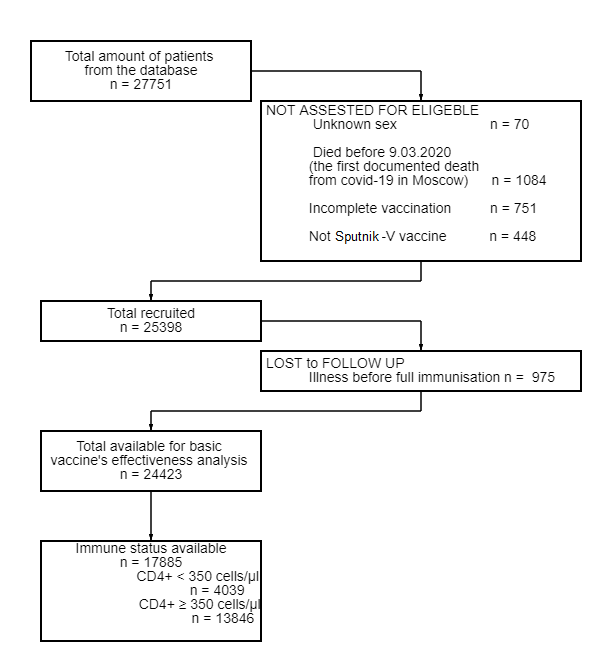
**

**Figure S2. The OR of COVID-19 infection probability associated with modification of different risk factors during the analyzed period.**

**
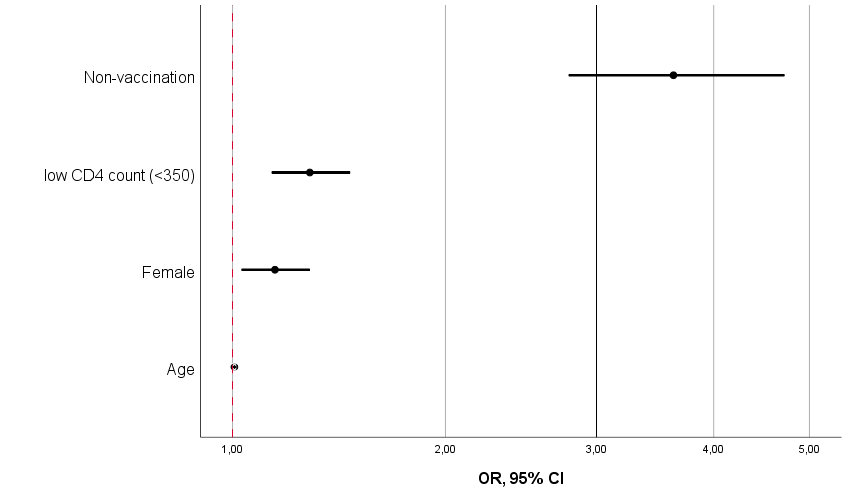
 Table 1. General characteristics of HIV+ individuals from the Moscow COVID-19 Vaccination and COVID-19 incidence Registries (patients on file at AIDS Moscow City Center).**

| **Sample characteristics** | **Vaccinated, n=2543 (10**·**4%)** | **Unvaccinated, n=17592 (72**·**0%)** | **Uncompleted vaccination *, n=4288 (17**·**5%)** | **p** |
| --- | --- | --- | --- | --- |
| Mean age  (M±SD, 95% CI) | 44·69±10·12  (44·30 – 45·09) | 41·50±10·11  (41·35 – 41·65) | 41·65±8·28  (41·40 - 41·90) | <0·001 Welch's T-test) |
| Females | 762 (30·0%) | 7 883 (44·8%) | 1 798 (41·9%) | <0·001 (chi square) |
| Males | 1 781(70·0%) | 9 709 (55·2%) | 2 490 (58·1%) |  |
| CD4+ (Me[IQR]) cells/µl  (n=17 885, 73·2%) | n=2 198 (86·4%)  639[484 - 821] | n=12 134 (69·0%)  526[334 - 730] | n=3 553 (82·9%)  586[424 - 774] | <0·001 (Mann - Whitney test) |
| СD4+ ≥350 cells/µl (n=13 846) | 1 967 (89·5%) | 8 883 (73·2%) | 2 996 (84·3%) | <0·001 (chi square) |
| СD4+ <350 cells/µl (n=4039) | 231 (10·5%) | 3 251 (26·8%) | 557 (15·7%) |  |

*data of patients with incomplete vaccination were not considered in group comparison

**Table 2. Characteristics of patients receiving ART in subgroups by CD4+ T-cells count (only fully immunized and unvaccinated were considered).**

| **Patients’ status** | **СD4+ ≥ 350 cells/µl** | |  | **СD4+ < 350 cells/µl** | |  |
| --- | --- | --- | --- | --- | --- | --- |
|  | **Vaccinated** | **Unvaccinated** | *P*<0·001  OR = 3·29 (2·51 – 4·30) | **Vaccinated** | **Unvaccinated** | *P*=0·002  OR = 2·53  (1·40 – 4·60) |
| With prior history of COVID-19 | 51  (2·6%) | 779  (9·3%) |  | 11  (4·8%) | 352  (11·7%) |  |
| No prior history of COVID-19 | 1 916  (97·4%) | 7 573 (90·7%) |  | 220  (95·2%) | 2 645 (88·3%) |  |

**Table 3. Typical for COVID-19 chest CT imaging features with extents of pulmonary involvement in HIV+ on ART depending on СD4+ Т-cells count.**

|  | **СD4+ ≥ 350 cells/µl** | | | **СD4+ <350 cells/µl** | | |
| --- | --- | --- | --- | --- | --- | --- |
|  | **Vaccinated**  **(documented n=15)** | **Unvaccinated**  **(documented n=334)** | **P** | **Vaccinated**  **(documented n=6)** | **Unvaccinated**  **(documented n=228)** | **P**  **(Fischer’s Exact test)** |
| **CT 0** | 7 (46·7%) | 76 (22·8%) | 0·043 | 1 (16·7%) | 55 (24·1%) | 0·916 |
| **CT 1** | 6 (40·0%) | 197 (59·0%) |  | 4 (66·7%) | 94 (41·2%) |  |
| **CT 2** | 0 (0·0%) | 45 (13·5%) |  | 1 (16·7%) | 39 (17·1%) |  |
| **CT 3** | 2 (13·3%) | 13 (3·9%) |  | 0 (0·0%) | 25 (11·0%) |  |
| **CT 4** | 0 (0·0%) | 3 (0·9%) |  | 0 (0·0%) | 15 (6·6%) |  |

**Table 4. Overall vaccine effectiveness in the entire group of HIV+, receiving ART.**

| **Patient cohorts** | **Vaccinated** | **Unvaccinated** |
| --- | --- | --- |
| COVID-19 illness | 71 (2·8%) | 1354 (8·2%) |
| No Covid-19 illness | 2472 (97·2%) | 15252 (91·8%) |
| Prior history of Covid-19 | 0 | 986 |
| Excluded from calculation due to incomplete immunization | 4288 | |
| Epidemiological effectiveness | 76·33% (95% CI: 69·84% - 81·43%) р<0·001 (chi square test) | |

**Table 5. Vaccine effectiveness among HIV+ in subgroups by CD4+ counts.**

| **CD4+ count** | **CD4+ < 350, n=4039** | | | **CD4+ >=350, n=13846** | | |
| --- | --- | --- | --- | --- | --- | --- |
| Patient cohorts | **Vaccinated** documented (n=231) | **Unvaccinated** documented (n= 2997) | P (chi square test) | **Vaccinated** documented (n=1967) | **Unvaccinated** documented (n= 8352) | P (chi square test |
| COVID-19 illness | 11  (4·8%) | 352  (11·7%) | 0·002 | 51  (2·6%) | 779  (9·3%) | <0·001 |
| No Covid-19 illness | 220 (95·2%) | 2645  (88·3%) |  | 1916  (97·4%) | 7573  (90·7%) |  |
| Prior history of Covid-19 | 0 | 254 |  | 0 | 531 |  |
| Excluded from calculation due to incomplete immunization | 352 | | | 779 | | |
| Epidemiological effectiveness | 73·15%  (50·27% – 85·50%) | | | 79·42%  (72·54% – 84·57%) | | |

**Table 6. Overall epidemiological effectiveness of vaccination against original and delta variants.**

| Period | 15 March – 15 May (circulation of original variant) | 1 June – 31 July  (circulation of delta variant) |
| --- | --- | --- |
| Number of vaccinated with *no prior history* of COVID-19 infection | 1257 | 2543 |
| Number of unvaccinated with *no prior history* of COVID-19 infection | 21193 | 15882 |
| Number of COVID-19 breakthrough cases among vaccinated, n | 8 | 59 |
| Number of COVID-19 cases among unvaccinated, n | 305 | 630 |
| Number of total COVID-19 cases before the beginning of analyzed period (regardless the immunization status) | 1308 | 1722 |
| Number of excluded from calculation due to incomplete immunization | 665 | 4288 |
| VE, % (95% CI) | 71·74% (42·85% – 86·03%) | 59·77% (47·28% – 69·30%) |

**Table 7. Impact of patients’ immune status on vaccine effectiveness during two time periods.**

| **Time period** | **15 March – 15 May** | | **1 June – 31 July** | |
| --- | --- | --- | --- | --- |
| **Immune status of HIV+ on ART** | **CD4+ <350 cells/µl** | **CD4+≥350 cells/µl** | **CD4+ <350 cells/µl** | **CD4+ ≥350 cells/µl** |
| Number of vaccinated with *no prior history* of COVID-19 infection | 111 | 961 | 227 | 1961 |
| Number of unvaccinated with *no prior history* of COVID-19 infection | 3501 | 116761 | 2774 | 7961 |
| Number of COVID-19 breakthrough cases among vaccinated, n | 3 | 4 | 7 | 45 |
| Number of COVID-19 cases among unvaccinated, n | 92 | 168 | 129 | 388 |
| Number of total COVID-19 cases before the beginning of analyzed periods (regardless the immunization status) | 361 | 695 | 477 | 922 |
| Number of excluded from calculation due to incomplete immunization | 66 | 514 | 557 | 2996 |
| VE, % (95% CI) | 33·47%  (-113·50% – 79·27%) | 81·17%  (49·13% – 93·03%) | 55·05%  (2·59% – 79·26%) | 65·34%  (52·61 – 74·66%) |

**Table 8. Impact of patient’s immune status on vaccine effectiveness in terms of protection from hospitalization during two time periods.**

| **Time period** | **15 March - 15 May** | | **1 June – 31 July** | |
| --- | --- | --- | --- | --- |
| **Immune status of HIV+ on ART** | **CD4+ <350 cells/µl** | **CD4+≥350 cells/µl** | **CD4+ <350 cells/µl** | **CD4+ ≥350 cells/µl** |
| **Number of vaccinated with *no documented COVID-19 illness*** | 111 | 961 | 227 | 1961 |
| **Number of unvaccinated with *no documented* COVID-19 illness (including immune stratum)** | 2201 | 7051 | 1755 | 4796 |
| **Number of hospitalized among vaccinated, n** | 1 | 0 | 3 | 6 |
| **Number of hospitalized among unvaccinated, n** | 58 | 54 | 62 | 74 |
| **VE, % (95% CI)** | 64·82% (-156·32% – 95·17%) | 100% | 59·92%  (-28·74% – 87·52%) | 75·77%  (44·25% – 89·47%) |

**Table 9. Impact of patient’s immune status on vaccine effectiveness against severe disease during two time periods.**

| **Time period** | **15 March - 15 May** | | **1 June – 31 July** | |
| --- | --- | --- | --- | --- |
| **Immune status of HIV+ on ART** | **CD4+ <350 cells/µl** | **CD4+≥350 cells/µl** | **CD4+ <350 cells/µl** | **CD4+≥350 cells/µl** |
| **Number of vaccinated with *no documented COVID-19 illness*** | 111 | 961 | 227 | 1961 |
| **Number of unvaccinated with *no documented* COVID-19 illness (including immune stratum)** | 2201 | 7051 | 1755 | 4796 |
| **Number of hospitalized among vaccinated, n** | 1 | 0 | 2 | 1 |
| **Number of hospitalized among unvaccinated, n** | 28 | 32 | 27 | 43 |
| **VE, % (95% CI)** | 27·14%  (-440·48% – 90·18%) | 100% | 38·64%  (-159·75% – 85·51%) | 93·05%  (49·51% – 99·04%) |

**Appendix**

**Supplementary tables**

**Table S1. Patients’ demographic data by time-periods.**

|  | **Sex** | **15 March – 15 May** | **1 June – 31 Juy** |
| --- | --- | --- | --- |
| **Vaccinated** | Males | 383  (79·6%) | 1 106  (73·4%) |
|  | Females | 98  (20·4%) | 400  (26·6%) |
| **Unvaccinated** | Males | 11 880  (56·0%) | 8 809  (55·3%) |
|  | Females | 9 316  (44·0%) | 7 118  (44·7%) |
| ***P* value** |  | <0·001* (chi square) | <0·001* (chi square) |

** - statistically significant difference (p<0·05)*
